# New Strategy to Preserve Phosphate by Ionic Liquid Matrices in Matrix-Assisted Laser Desorption/Ionization: A Case of Adenosine Nucleotides

**DOI:** 10.3390/molecules25051217

**Published:** 2020-03-08

**Authors:** Chih-Hao Lin, Chuping Lee, Yu-Cheng Wu, I-Chung Lu

**Affiliations:** 1 Department of Chemistry, National Chung Hsing University, Taichung City 40227, Taiwan; ctes955113@gmail.com (C.-H.L.); ff40617tw@gmail.com (Y.-C.W.); 2 Department of Applied Chemistry, National Chiayi University, Chiayi City 60004, Taiwan; cplee@g.ncyu.edu.tw

**Keywords:** ionic liquids, MALDI, ionic liquid matrix, soft ionization, nucleotides, triphosphate, Adenosine, AMPK, ATP

## Abstract

Adenosine -5′-triphosphate (ATP) plays a valuable role in metabolic activity to produce adequate energy in a biosystem. A high ATP/AMP ratio has a correlation with diabetes that induces suppression of AMP-activated protein kinase (AMPK). Matrix-assisted laser desorption/ionization (MALDI)–mass spectrometry (MS) has outstanding potential in determining the ratio of several types of adenosine phosphates in a sample to rapidly understand the primary energy transfer in metabolism. Although MALDI is viewed as a soft ionization technique for MS analysis, excess photon energy might crack the phosphate bonds leading to misinterpretation of the ATP level. In this work, ionic liquid matrices (ILMs) were employed to reduce fragmentation and increase the detection efficiency during the MALDI process. This study demonstrated for the first time that 2,5-dihydroxybenzoic acid pyridine (DHBP) is one of the most effective matrices for further quantitative analysis of adenosine nucleotides. This systematic screening of ILMs also enhances the fundamental understanding of MALDI.

## 1. Introduction

Adenosine-5′-triphosphate (ATP) has been studied as a “molecular currency,” which stores and transfers chemical energy in living cells [1]. It is essential as a metabolic regulatory parameter between anabolism and catabolism. ATP is composed of adenine and ribose with three phosphates, of which two high-energy phosphate bonds can be hydrolyzed to adenosine-5′-diphosphate (ADP) and adenosine -5′-monophosphate (AMP) to release energy. Most cells maintain the intracellular ATP/ADP ratio at 10:1. ATP level balance is closely related to energy homeostasis [2,3]. Recently, numerous studies have indicated that the level of ATP could influence metabolism activities in various cancer cells [4,5]. For example, AMP-activated protein kinase (AMPK) is a sensor of cellular energy change, which is activated by increasing AMP/ATP or ADP/ATP ratios [6,7,8,9]. Dysfunction of ATP synthesis and consumption can cause AMPK maladjustment [10]. Therefore, it is essential to determine the ratio between AMP, ADP, and ATP in a biological system to understand primary energy transfer in metabolism. Ambiguous quantitative analysis may lead to misinterpreted values and thus distort real events in the biosystem.

Electrospray ionization (ESI) and matrix-assisted laser desorption/ionization (MALDI), two of the most popular soft ionization techniques, are widely used for the analysis of biomolecules [11,12,13,14]. A soft ionization process provides the opportunity to observe intact biomolecules in a mass spectrum rather than the fragments of ions struck by high energy. Although it is generally believed that the ionization process of ESI is milder than that of MALDI, by avoiding the fragmentation of labile analytes, MALDI has the advantages of straightforward sample preparation, high throughput, and low interference from background salts. Therefore, it is widely used in various research fields and has unique advantages. Some small molecules are mixed with an analyte as the MALDI matrix to absorb photon energy from a pulsed laser, and then the analyte is desorbed into the gas phase and ionized. Compared with direct high-energy irradiation by lasers or particle beams [15,16]. MALDI reduces the requirement of laser energy through the matrix as a medium. The milder ionization process of MALDI provides the opportunity to observe the original structures of the analyte without breaking it during the desorption and ionization process.

Although conventional MALDI is considered a soft ionization technique, many studies have revealed that labile molecules, such as glycans containing sialic acid, phosphate, sulfate, acetyl peptides, gangliosides, and polymers readily dissociate into fragments during MALDI [17,18,19,20,21,22]. Consequently, several modified methods or new matrices have been developed to overcome the cracking problem in the ionization process. For instance, ionic liquids (ILs) were used as matrices to not only provide homogenous morphology but also preserve the intactness of ganglioside and phospholipid ions [23]. In another study, nanomaterials modified the MALDI sample plate to increase sensitivity and reproducibility for glucans (pullulan and dextran) [24,25]; moreover, frozen MALDI was employed, in which the solution temperature decreased to 100 K, making ganglioside and heparin ions remained intact [26,27,28]. The aforementioned ionization techniques might successfully reduce fragmentation, but the spot-to-spot reproducibility of the MALDI sample remains a challenge for quantitative analyses. ILs have many advantages in various applications, including application in MALDI– mass spectrometry (MS) [29,30]. The substitution of the conventional MALDI matrix with ILs leads to substantial benefits. IL matrices (ILMs) exhibit characteristics such as vacuum stability, homogeneous morphology, high reproducibility during sampling, and minimal fragment ion production [31]. ILMs allow both qualitative and quantitative measurement of MALDI–MS and are therefore widening the field of applications for this analytical method [32]. Moreover, an ILM can be conveniently operated in a commercial MALDI–time-of-flight (TOF), which is suitable for this study.

High-performance liquid chromatography (HPLC)/ESI–MS is commonly used for quantitative analysis of biomolecules. However, the platform is unsuitable for the quantitative analysis of adenosine nucleotides because of the loss of phosphate [33]. The fragment ions of ATP would superimpose on the ADP or AMP signal to hamper the quantitative identification of adenosine nucleotides in the applications. As a tool for the quantitative analysis of adenosine nucleotides, MALDI–TOF may also misinterpret data as a result of the bond cleavage at phosphate ester/anhydride linkages [34,35,36]. Some studies have replaced conventional MALDI matrices, such as 2,5-dihydroxybenzoic acid (DHB) and α-cyano-4-hydroxycinnamic acid (CHCA) with novel organic compounds, nanoparticles, or ILs for higher sensitivity and lower background interference [37,38,39,40]. However, the matrices generate a certain percentage of fragments, which obstruct further quantitative analysis. Thus, this study demonstrated a strategy for discovering a MALDI matrix that can analyze adenosine nucleotides based on ILs. We also analyzed the mixtures of ATP, ADP, and AMP by using the ILMs to demonstrate the further ability of quantitative analysis. A series of CHCA ILs and DHB ILs were used as matrices for adenosine analysis. The relative photon absorbance, spot-to-spot variation, and suppression effect of the ILMs were studied. The experimental results were used as a model to confirm the excellent capability of ILs to serve as effective MALDI–MS matrices.

## 2. Results

**Scheme 1:**
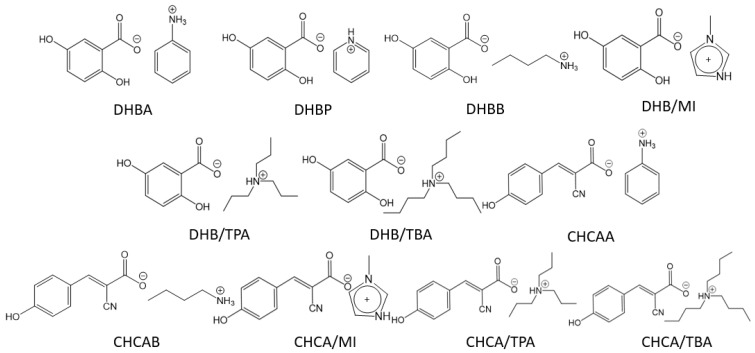
The molecular formula of synthesized DHB and CHCA ILs.

A wide variety of ionic matrices have been used as MALDI matrices for different applications. In this study, the conventional matrices DHB and CHCA were employed for comparison with their derived ILs. These ILs were synthesized, as illustrated in Scheme 1. At room temperature, DHBB (DHB-butylamine), DHB/MI (DHB-1-methylimidazole), DHB/TPA (DHB-tri-n-propylamine), CHCA/MI(CHCA-1-methylimidazole), CHCA/TBA (CHCA-tri-n-butylamine), and CHCA/TPA(CHCA-tri-n-propylamine) are liquid and DHBA (DHB-aniline), DHBP (DHB-pyridine), DHB/TBA (DHB-tri-n-butylamine), CHCAA(CHCA-aniline), and CHCAB (CHCA-butylamine) are solid. The melting points of all these matrices were accordingly determined and provided in Table S1 (Supplementary Information). In this study, the MALDI–MS spectra of ATP, ADP, and AMP were demonstrated by the aforementioned matrices.

### 2.1. Fragmentation Yield

**Figure 1:**
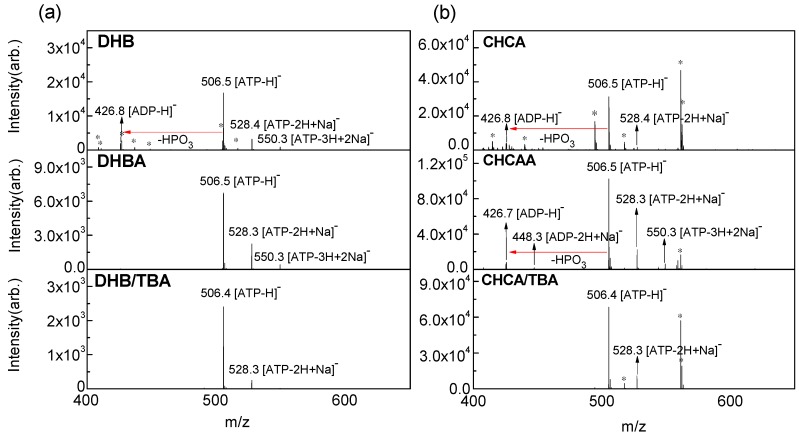
MALDI mass spectra of ATP (1 nmol) in one spot with the matrix of (**a**) DHB compared with DHBA, and DHB/TBA ILs and (**b**) CHCA compared with CHCAA and CHCA/TBA in a negative reflectron mode. The molar ratio of the matrix-to-analyte was approximately 500:1. The relative laser energy was 95% and 75% for each group, respectively. The star symbols denote the signals of the matrix background.

**Figure 2:**
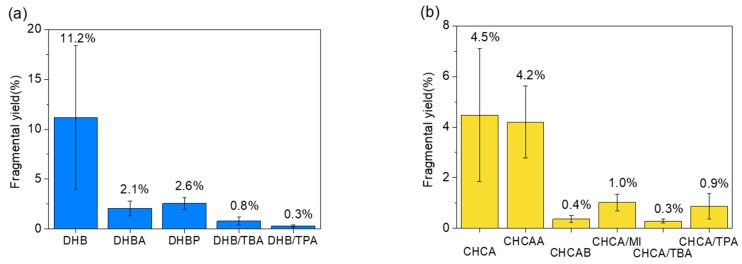
The fragmentation yield of ATP ion signal in various matrices: (**a**) DHB ILs and (**b**) CHCA ILs. The error bar of each data set was calculated by the intensity values (n = 20) of each matrix.

To identify the IL as the most suitable matrix for quantitative analysis, the critical conditions of MALDI, such as matrix-to-analyte ratio and relative absorbance, were optimized for both positive and negative ions. The mass spectrum (MS) of ATP was performed using conventional matrices and ILMs to test the sensitivity of our target. Comparing the details of MS for ATP in positive (Figure S1) and negative (Figure S2) ions, the mass spectra in the negative mode presented less matrix background, which is relevant to evaluating the fragmentation yield of ATP. However, the class of matrix resulted in distinct spectral qualities. The signal intensity was higher when using CHCA and its related ILs, but the background noise was also higher. The system using DHB and its related ILs exhibited a clear signal of ATP, whereas the use of DHBB and DHB/MI resulted in poor sensitivity. Figure 1 lists some of the comparisons of MALDI–MS of ATP between conventional matrices and ILMs. The deprotonated [ATP-H]^−^ (*m*/*z* 506.5) dominated all of the spectra. In addition to the deprotonated molecules, signals for sodium adducts could also be observed in negative ion mode. Both [ATP-2H+Na]^−^ (*m*/*z* 528.3) and [ATP-3H+2Na]^−^ (*m*/*z* 550.3) exhibited minor peaks corresponding to ATP in the spectrum. The ion signals of the matrix were studied and then labelled in each spectrum (Figure S3). In some spectra, [ADP-H]^−^ (*m*/*z* 426.8) was present, which accompanied by the loss of phosphate (HPO_3_) from [ATP-H]^−^ because of the extra energy leading to fragmentation during the ionization process. Compared with the conventional matrix, ILMs were used with relative success and reduced the fragmentation effectively, except DHBB and DHB/MI, which failed to detect ATP. The fragmentation yields of [ATP-H]^−^ for definite matrices (presented in Figure 2) illustrate their “softness.” The yields were estimated by summarizing the ion signal of [ADP-H]^−^ and [ADP-2H+Na]^−^ and then dividing by the total ion signal corresponding to the analyte. The conventional solid matrices presented higher fragmentation yields, illustrating their “hard” characteristics in the ATP ionization process. Furthermore, they exhibited a higher relative standard deviation (RSD) of the ion signal due to low homogeneity, which will be discussed in the subsequent section.

In summary, the ILMs exhibited much lower fragmentation yields than the conventional solid matrix. DHB/TBA, DHB/TPA, CHCA/TBA, and CHCA/TPA were apparently superior to the others to serve as a soft MALDI matrix with fragmentation yields of 0.80, 0.29, 0.28, and 0.87 percent, respectively. In addition to the negligible fragmentation (<1%), the aforementioned ILMs did not produce intense cluster ions or matrix ions in MALDI–MS, which facilitates interpretation. The lower fragmentation of analyte ions is a critical advantage for quantitative analysis because the interference from the fragmentation often leads to interpretation difficulty for mass spectra of ATP, ADP, and AMP. Moreover, the detection efficiency of the analyte is the main parameter to evaluate applicable matrices. The conventional matrices and ILMs were examined by the detection efficiency of ADP and AMP shown in Figures S4 and S5. Although DHB/TBA and DHB/TPA demonstrated extremely low fragmentation yield to ATP, their detection efficiency to AMP was insufficient for further applications (Figure S5). CHCA and related ILMs demonstrated favorable detection efficiency of ATP and ADP. However, most of them were not successful in detecting AMP, except CHCA and CHCA/TPA (Figure S5) and did not appear to be suitable candidates for further quantitative analysis.

### 2.2. Possible Ionization Mechanism of ILM

Although UV-MALDI has been widely used in the mass analysis of biomolecules, its ionization mechanism remains unclear. The selection of a suitable matrix typically relies on trial and error or experience; the same applies to ILMs. Several mechanisms have been proposed to explain the ionization process in MALDI [41,42,43]. In these modes, photon energy absorption and proton transfer are the main concerns in the ionization mechanism. However, these proposed mechanisms were reported for conventional solid matrices but not necessarily valid for ILMs. In this section, we represented the UV absorption and molecular structure of ILMs to elucidate their characteristics.

**Figure 3:**
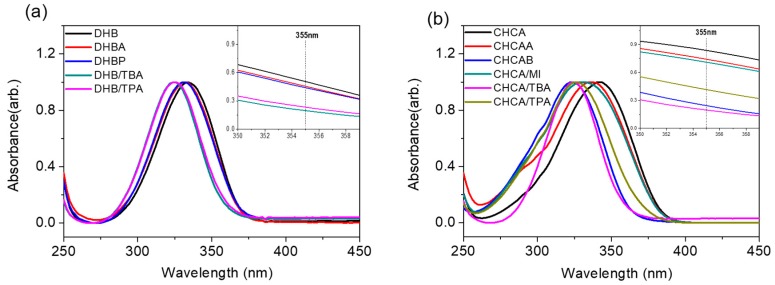
UV absorption spectrum of various matrices: (**a**) DHB and DHB ILs and (**b**) CHCA and CHCA ILs. All of the spectra were normalized by the highest absorption band.

**Scheme 2:**
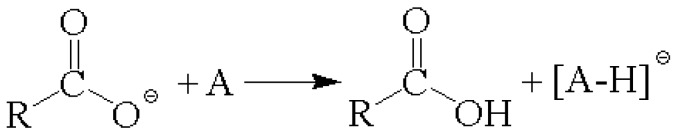
Proton transfer reaction between ILM and analyte.

The UV spectrum absorption profiles of conventional matrices and ILMs were shown between 250 and 450 nm (Figure 3). All the spectra were normalized by the highest absorption band to compare the band shift after being synthesized into ILs. The ILMs presented a blue shift to solid matrices, DHB and CHCA, but no significant changes to the profiles were observed. The UV spectrum provided by ILMs had lower absorbance at the required wavelength (355 nm) to generate a “colder” environment during the ionization process (Figure 3 inset). Studies have revealed that an increase in surface temperature can effectively enhance the proton transfer reaction in UV-MALDI [44]. Therefore, a decrease of the absorption cross-section would not only suppress the fragmentation but also decrease the ionization efficiency considerably. However, except for DHBB, DHB/MI, and DHB/TPA, most ILMs produced sufficient signal ions when ADP was used as the analyte (Figure S4). Because of the intrinsic carboxylate group in ILMs, the proton is transferred effectively from analyte to the carboxylate group in CHCA and DHB forming negatively analyte ions even if the thermal energy is lower than in conventional conditions (Scheme 2). However, the heat of deprotonation of carboxylate group in CHCA (M → [M − H]^−^ + H^+^) was 316 kcal/mol, which is smaller than that in DHB (325 kcal/mol) [28]. This suggests that the CHCA ILMs had a lower ability to remove a photon from the analyte when the acidity of the analyte was lower. Therefore, the detection efficiency of AMP was lower when using CHCA ILMs, which confirms the aforementioned deduction.

### 2.3. Homogeneity of Sample Preparations

**Figure 4:**
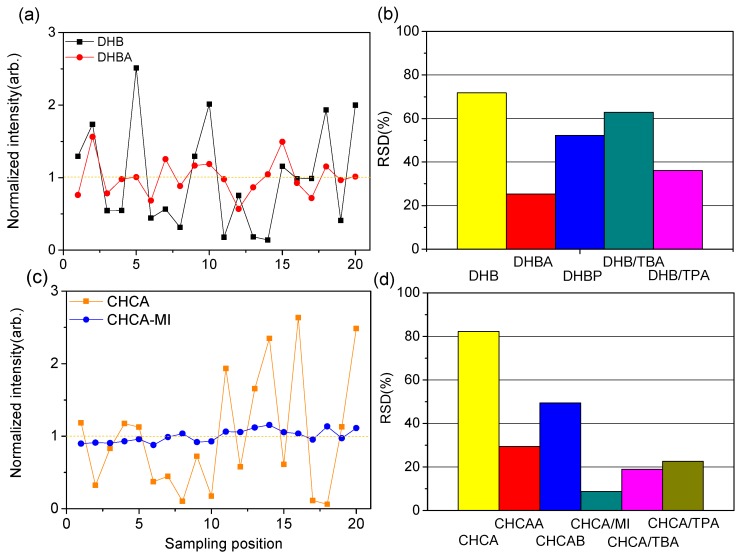
Plot of normalized [ATP-H]^−^ signal distribution of (**a**) DHB (black square) and DHBA IL (red circle) and (**c**) CHCA (orange square) and CHCA/MI IL (blue circle). The averaged RSD value in 20 different positions of (**b**) DHB ILs and (**d**) CHCA ILs. Each sampling spot was irradiated by 1000 laser shots.

The homogenous distribution of matrix and analyte molecules in the sample spot is a critical consideration in MALDI–MS. This is particularly crucial for quantitative measurements in which numerous unsuccessful shots have a negative influence on the measurement. Some studies have compared the homogenous characteristics between conventional matrices and ILMs to demonstrate the advantages in spot-to-spot reproducibility for quantitative studies [23]. In this work, the spot-to-spot variation of ILMs was compared to select a suitable candidate for further quantitative analysis (Figure S6). The signal intensity of [ATP-H]^−^ was collected from 20 random sampling positions. The signal reproducibility was demonstrated by their RSD values calculated from the variation of signal intensities (listed in Figure 4). Among the conventional matrices, the RSD was 72% for DHB and 81% for CHCA; sample preparation using solid matrices represented the “hot spots” phenomenon in both DHB and CHCA. However, most of the ILMs exhibited lower signal variation than conventional matrices. Notably, the liquid-like ILMs (DHB/TPA, CHCA/MI, CHCA/TPA, and CHCA/TBA) displayed better homogeneity compared with others ILMs (Figure 4b,d).

### 2.4. Quantitative Analysis of ATP

To identify the relative ratio of ATP, ADP, and AMP in a biological sample, considering the suppression effect between the analytes in a mixture is essential. The degree of suppression effect can be observed by comparing ion intensity between the spectra of a single component and the mixture. Because of the high fragmentation yield of CHCAA, high signal variation of CHCAB (Figure 4d) and insufficient detection efficiency intensity of DHB/TBA, DHB/TPA, and CHCA/TBA in AMP (Figure S5), only four of ILMs were selected for examining the suppression effect (Figures S7–S10). In general, the detection efficiency of AMP in MALDI is relatively low, which also presented a considerable suppression effect in these four matrices. Considering the fragmentation yield, detection efficiency, and homogeneity in the aforementioned experimental results, DHBP and CHCA/TPA were selected as the two most suitable candidates for the final evaluation.

**Figure 5:**
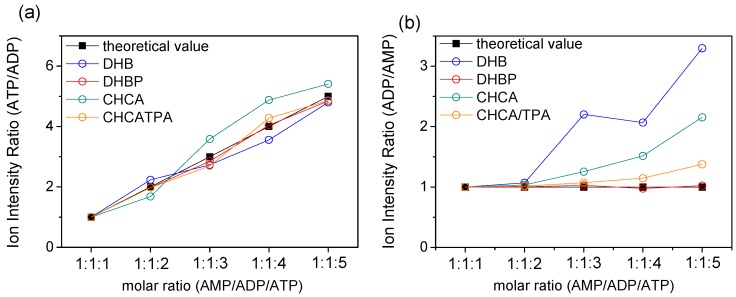
The ion intensity ratios of theoretical and experimental results. The molar ratios were 1:1:1, 1:1:2, 1:1:3, 1:1:4, and 1:1:5 for AMP, ADP, and ATP, respectively. The concentration of ATP was gradually increased, whereas concentrations of AMP and ADP were fixed. The ratios were all normalized by 1:1:1 point: (**a**) ratio of ATP/ADP from 1:1:1 to 1:1:5 and (**b**) ratio of ADP/AMP from 1:1:1 to 1:1:5 (n = 8).

AMP, ADP, and ATP were premixed in the stock solution at molar ratios of 1:1:1, 1:1:2, 1:1:3, 1:1:4, and 1:1:5. Figure 5 reports the ratio of the signal intensity for ATP/ADP and ADP/AMP using conventional matrices and ILMs DHBP and CHCA/TPA. The black square represents the molar ratio of the analyte in-stock solution, which could be connected to a straight line. The experimental results were demonstrated by hollow circles distinguished by color codes. For further comparison, the ratios for distinct matrices were normalized by the data point of molar ratio 1:1:1. Figure 5a presents the ratio of ATP/ADP, and Figure 5b presents the ratio of ADP/AMP in the five aforementioned mixtures. In the case of prepared mixtures, the proportion of ADP/ATP increased with different samples, but the proportion of ADP/AMP was kept constant in this experiment.

Both Figure 5 and Figure S11 demonstrated the perfect fit to theoretical values when DHBP was employed as the MALDI matrix. When the experimental data deviated below the straight line, it suggested that an extra ion signal of ADP was cracked from ATP during the ionization process (Figure 5a). The conventional matrices, DHB (blue circle) and CHCA (green circle) presented this characteristic due to the “hard” ionization environment. However, most experimental data deviated above the straight line in Figure 5b, which suggests that the detection efficiency of AMP decreased more than that of ADP in the mixture. A critical concern is the varying degree of changes in the detection efficiency of mixtures during MS analysis. Herein, the ILM DHBP demonstrated excellent performance in the definite detection efficiency between single components and mixtures. Moreover, DHBP is sufficiently “cold” enough to reduce fragmentation of the analyte during the MALDI process.

In conclusion, we discovered that DHBP has an excellent capability to work in MALDI–MS for adenosine nucleotides analysis. This makes DHBP an ideal tool for screening biological systems and understanding primary energy transfer in metabolism.

## 3. Discussion

A novel strategy using ILs as MALDI matrices were studied for analyzing the adenosine nucleotides, ATP, ADP, and AMP. ILMs displayed more advantages than traditional MALDI matrices and could be applied in dried-droplet preparations in MALDI–MS analysis as well. The ILMs were kept under high vacuum conditions in the MS instrument; therefore, no manual selection of hot spots was necessary to yield MALDI–MS for an appropriate signal-to-noise ratio. The ILs could efficiently ionize adenosine nucleotides with only minor fragmentation, that characteristic provides a valuable tool to accurately interpret the results in energy metabolism activities.

In summary, we demonstrated that the IL DHBP is a highly suitable matrix for analyzing adenosine nucleotides preventing the fragmentation of phosphate group. Compared with the conventional matrix, DHBP has lower UV absorption at 355 nm, which represents a milder ionization process that reduces the fragmentation of phosphate bonds in ionization. Moreover, the intrinsic carboxylate group enhanced the proton transfer to produce the deprotonated anion of the analyte to compensate for the lack of thermal energy. Furthermore, the reproducibility of signal intensity was considerably improved by ILMs, as was the case with DHBP. Finally, the suppression effect was almost negligible when we analyzed a mixture. These features of DHBP provide the opportunity to analyze adenosine nucleotides quantitatively and rapidly by MALDI–MS. After optimization of parameters such as sample preparation procedure, pH, or solvent conditions, DHBP ILM holds much promise for future applications, especially for diabetes.

## 4. Materials and Methods

### 4.1. Materials

ATP-disodium salt (ATP-2Na) (99%), ADP-disodium salt (ADP-2Na) (≧95%), AMP-disodium salt (AMP-2Na) (99%), DHB (98%), CHCA (98%), and aniline were purchased from Sigma-Aldrich (St. Louis, MO, USA). Tri-n-butylamine and 1-methylimidazole were purchased from Alfa Aesar (Tewksbury, MA, USA). Butylamine and tri-n-propylamine were purchased from Acros Organics (Geel, Belgium). Pyridine was purchased from Showa (Saitama, Japan). Methyl alcohol (≥99.9%), ethyl alcohol (≥99.9%), and acetonitrile (≥99.9%) were purchased from Avantor (Radnor, PA, USA). The chemicals were used without further purification and pretreatment before experiments. Water was purified using a purification system (PORETECH, Ultrapure water system, Taiwan); all organic solvents were all HPLC grade.

### 4.2. Synthesis of ILMs and MALDI Sample Preparation

The stock solution of the conventional matrices was prepared with 1:3 (*v*/*v*) H_2_O/acetonitrile (1 M for DHB and 0.1 M for CHCA). The synthesis of ILM was prepared by dissolving 0.2 mmole DHB or CHCA in methanol. An equimolar amount of a series of amines (0.2 mmol) including aniline, pyridine, butylamine, 1-methylimidazole, tri-n-butylamine, and tri-n-propylamine were added to the matrix solution [45]. After mixing the solution on a vortex for 2–3 min, a vacuum oven was used to efficiently evaporate methanol and residual amine for 24 h.

The produced ILs were diluted with ethanol to a final concentration of 1 M (for liquid ILs) and 0.2 M (for solid ILs) as a stock solution. Stock solutions of analytes (2 mM) were prepared in deionized water. MALDI samples were mixed with stock solutions 1:1 and 5:1 (*v*/*v*) of the matrix and the analyte to molar ratios of matrix-to-analyte (M/A) between 500:1 and 50:1. Afterward, 1-3 μL of the mixture solution was spotted onto a stainless-steel MALDI plate by using the dried-droplet method.

### 4.3. Instrument

Bruker Ultraflex III TOF mass spectrometer (Bruker Daltonics, Gmbh, Germany) was employed in this study operating on reflectron mode. The Nd:YAG laser system was operated at 355 nm and 100-Hz repetition rate with appropriate relative laser energy (75–100%) and mass range. All mass spectra were acquired by summing 1000 single-shot mass spectra, unless stated otherwise.

The UV-Vis absorption spectra (200–600 nm) of the DHB, CHCA, or IL solutions were performed using a UV spectrophotometer (Hitachi, Westford, MA, USA, U-3010 UV-Visible, scanning spectrophotometer, double beam, 1 cm optical path length). All stock solutions (1 M for DHB, 0.1 M for CHCA, and 0.2–1 M for ILs) was diluted 1000 times before loading into the quartz cell.

## Supplementary Materials

The following are available online, Figure S1: MALDI mass spectra of ATP (1 nmol) in one spot with the matrix of (a) DHB ILs and (b) CHCA ILs in positive reflectron mode, Figure S2: MALDI mass spectra of ATP (1 nmol) in one spot with the matrix of (a) DHB ILs and (b) CHCA ILs in negative reflectron mode, Figure S3: MALDI mass spectra of matrix background for (a) DHB ILs and (b) CHCA ILs in negative reflectron mode, Figure S4: MALDI mass spectra of ADP (1 nmol) in one spot with the matrix of (a) DHB ILs and (b) CHCA ILs in negative reflectron mode, Figure S5: MALDI mass spectra of AMP (1 nmol) in one spot with the matrix of (a) DHB ILs and (b) CHCA ILs in negative reflectron mode, Figure S6: Plot of normalized [ATP-H]^−^ signal distribution of (a) DHB ILs and (b) CHCA ILs in 20 different positions of one sample spot on the MALDI metal plate, Figure S7: MALDI mass spectra of (a) AMP (1 nmol) (b) ADP (1 nmol) (c) ATP (1 nmol) (d) AMP:ADP:ATP = 1:1:1 (0.33 nmol for each one) with DHBA IL in negative reflectron mode, Figure S8: MALDI mass spectra of (a) AMP (1 nmol) (b) ADP (1 nmol) (c) ATP (1 nmol) (d) AMP:ADP:ATP = 1:1:1 (0.33 nmol for each one) with DHBP IL in negative reflectron mode, Figure S9: MALDI mass spectra of (a) AMP (1 nmol) (b) ADP (1 nmol) (c) ATP (1 nmol) (d) AMP:ADP:ATP = 1:1:1 (0.33 nmol for each one) with CHCA/MI IL in negative reflectron mode, Figure S10: MALDI mass spectra of (a) AMP (1 nmol) (b) ADP (1 nmol) (c) ATP (1 nmol) (d) AMP:ADP:ATP = 1:1:1 (0.33 nmol for each one) with CHCA/TPA IL in negative reflectron mode, Figure S11: Quantitative comparison between theoretical and experimental ratios of AMP, ADP, and ATP, Table S1: Melting points of conventional matrices (DHB and CHCA) and IL matrices measured by the Mel-Temp II Capillary Melting Point Apparatus (Laboratory Devices Inc., USA).
