# Supplementary material for: New Strategy to Preserve Phosphate by Ionic Liquid Matrices in Matrix-Assisted Laser Desorption/Ionization: A Case of Adenosine Nucleotides
Source: Molecules. 2020 Mar 8;25(5):1217. doi: 10.3390/molecules25051217 (PMC7179418; doi:10.3390/molecules25051217)
Supplement: Supplementary file 1 [file molecules-25-01217-s001.pdf]

(a) Positive DHB ILs

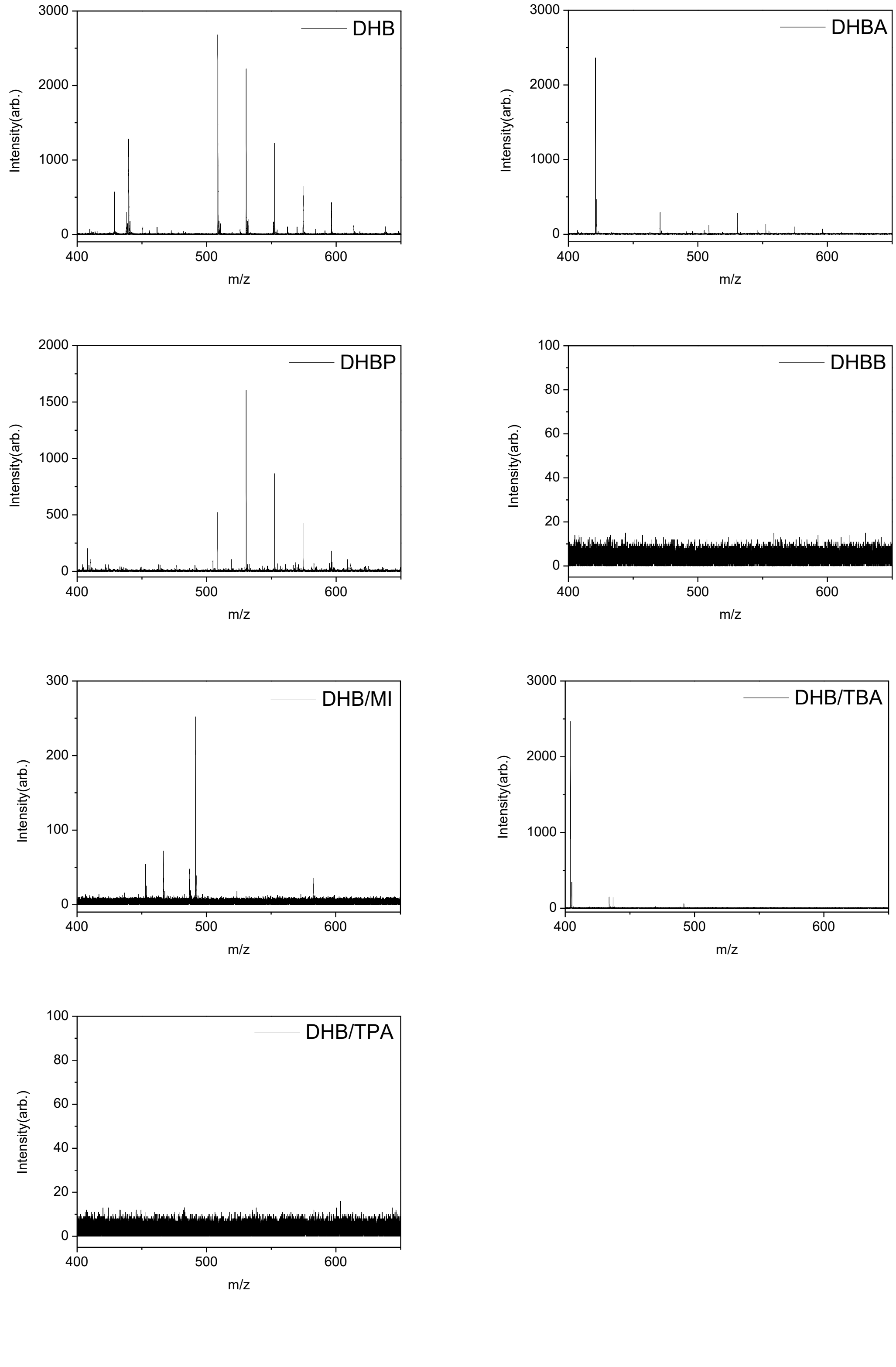

(b) Positive CHCA ILs

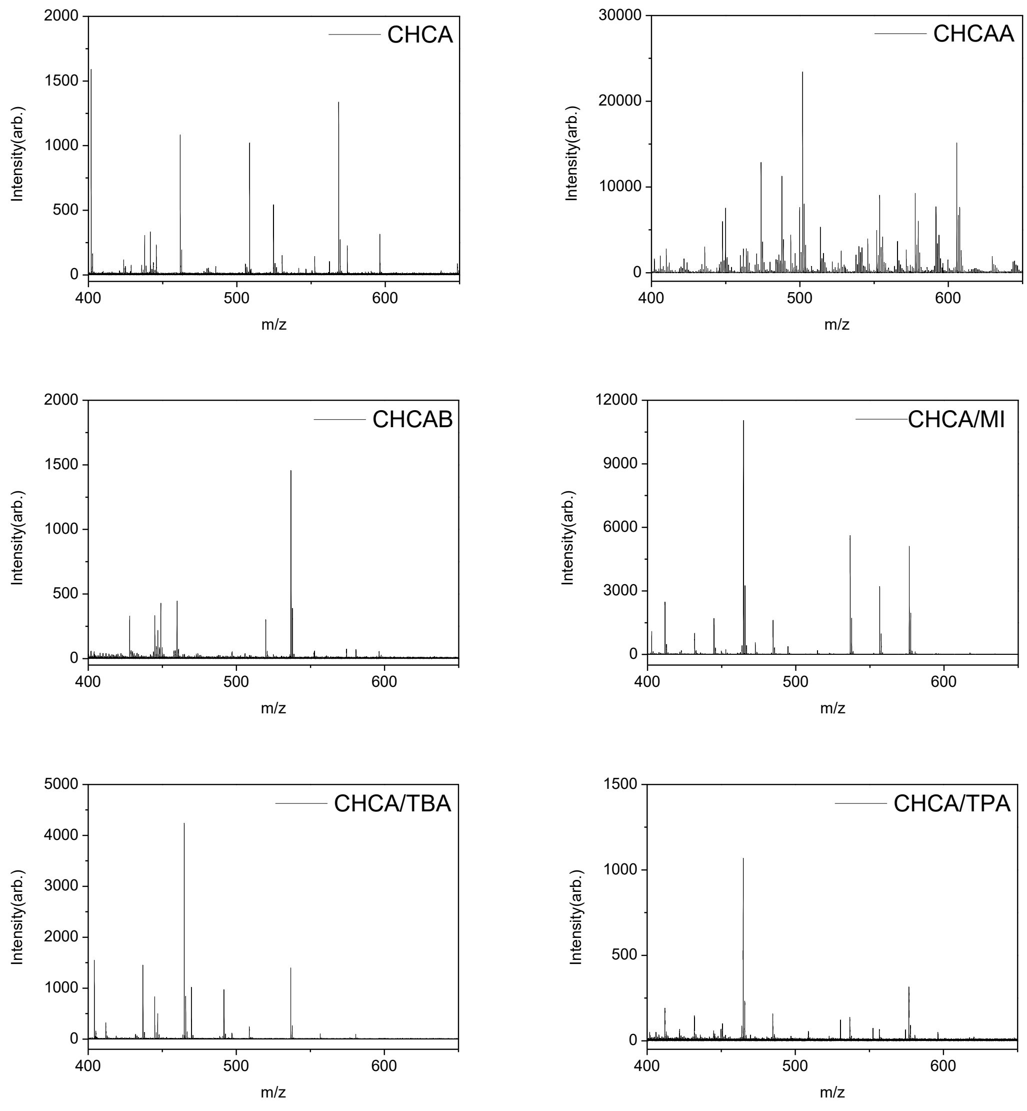

Fig S1. MALDI mass spectra of ATP (1 nmol) in one spot with the matrix of (**a**) DHB ILs and (**b**) CHCA ILs in positive reflectron mode. The molar matrix-to-analyte ratio was approximately 500:1. Laser energy was 85% and 65% for each group. 1000 laser shots randomly distributed in 20 different positions.

(a) Negative DHB ILs

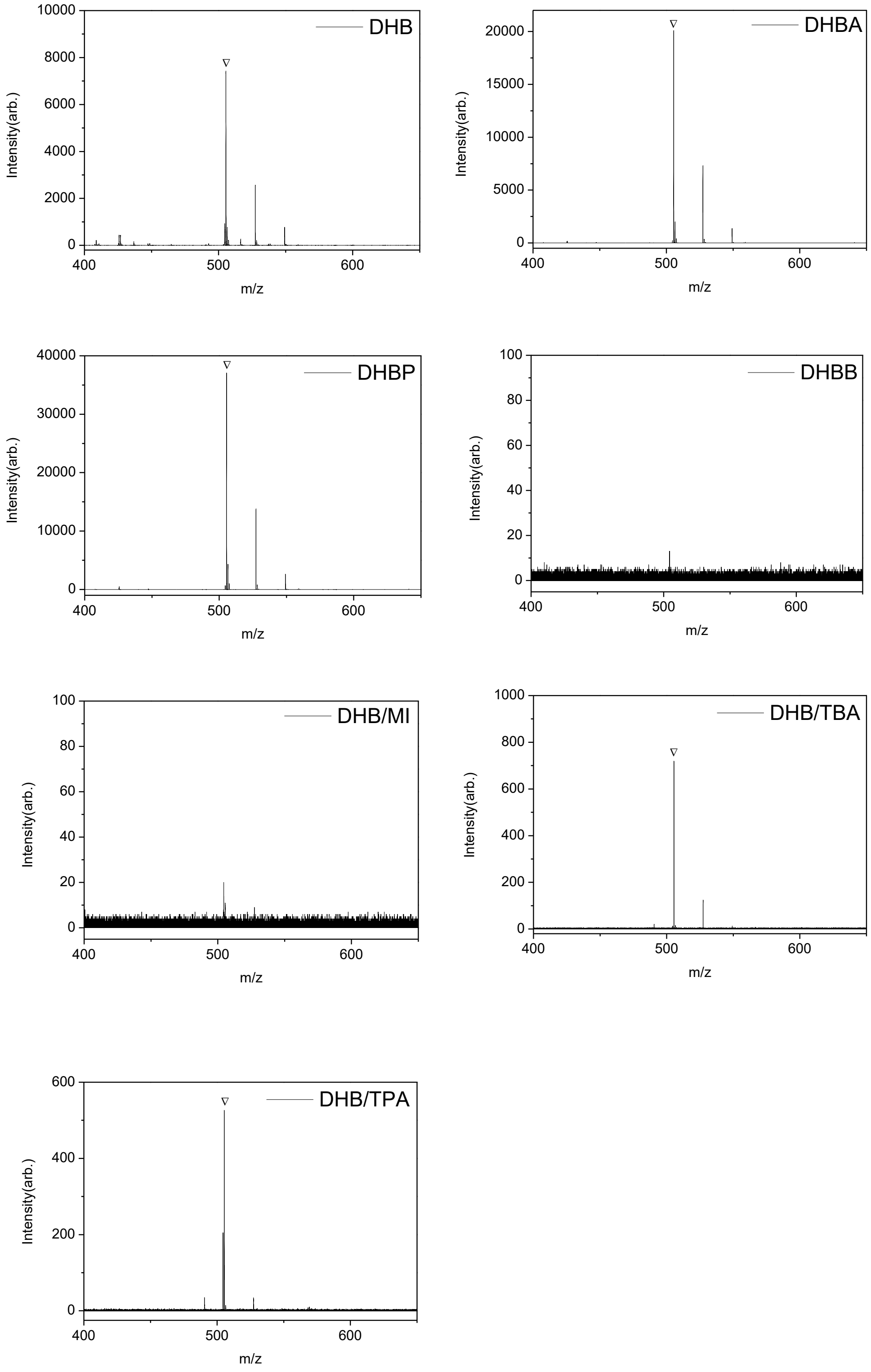

(b) Negative CHCA ILs

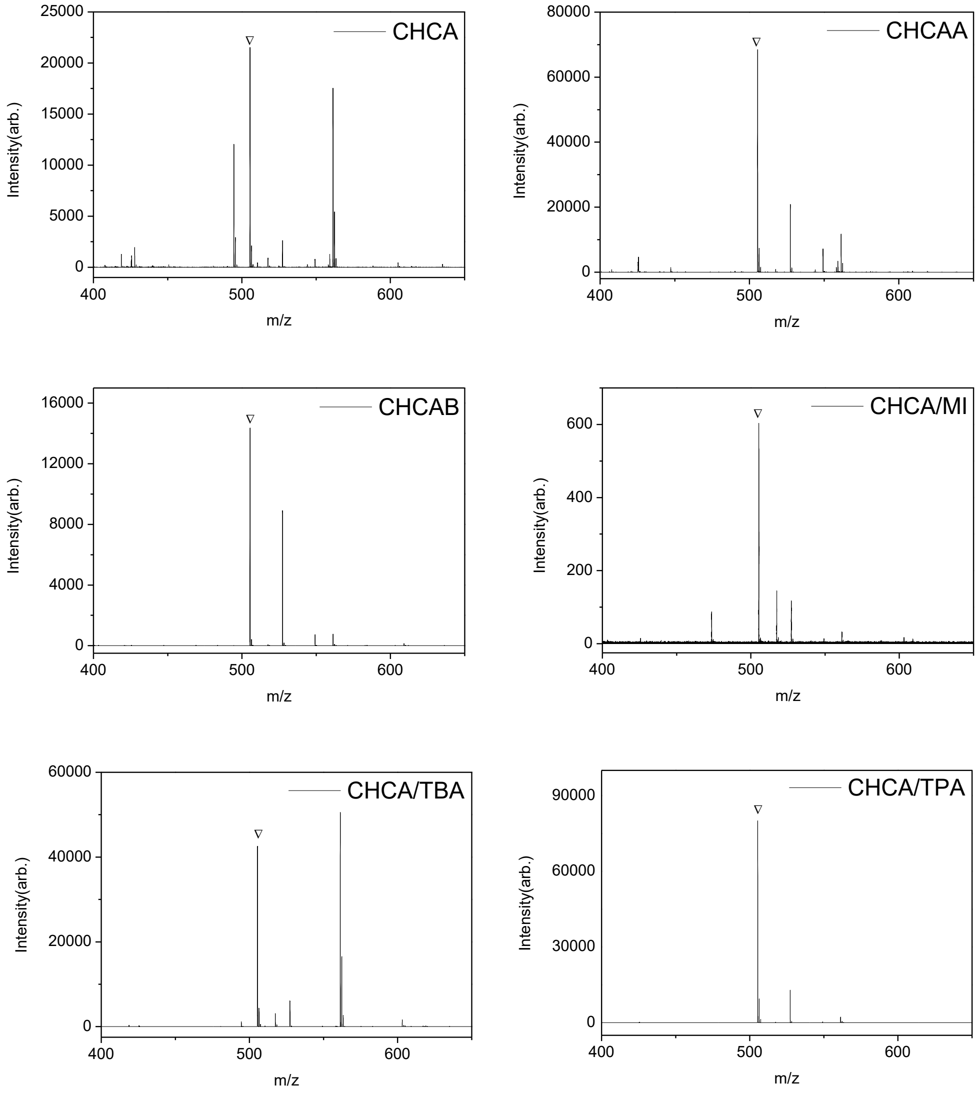

Fig S2. MALDI mass spectra of ATP (1 nmol) in one spot with the matrix of (**a**) DHB ILs and (**b**) CHCA ILs in negative reflectron mode. The molar matrix-to-analyte ratio was approximately 500:1. Laser energy was 95% and 75% for each group. 1000 laser shots randomly distributed in 20 different positions.

(a) Negative DHB ILs

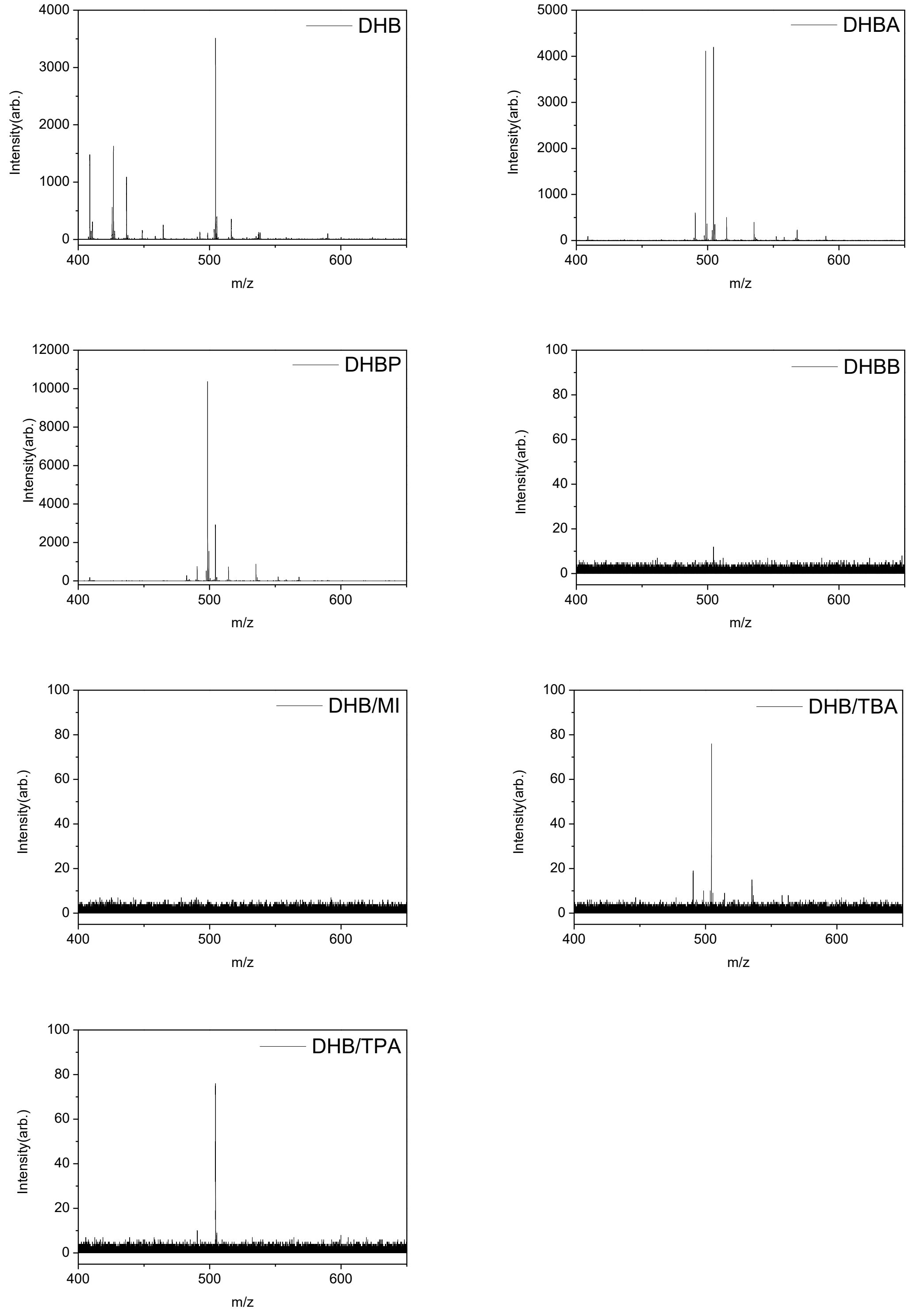

(b) Negative CHCA ILs

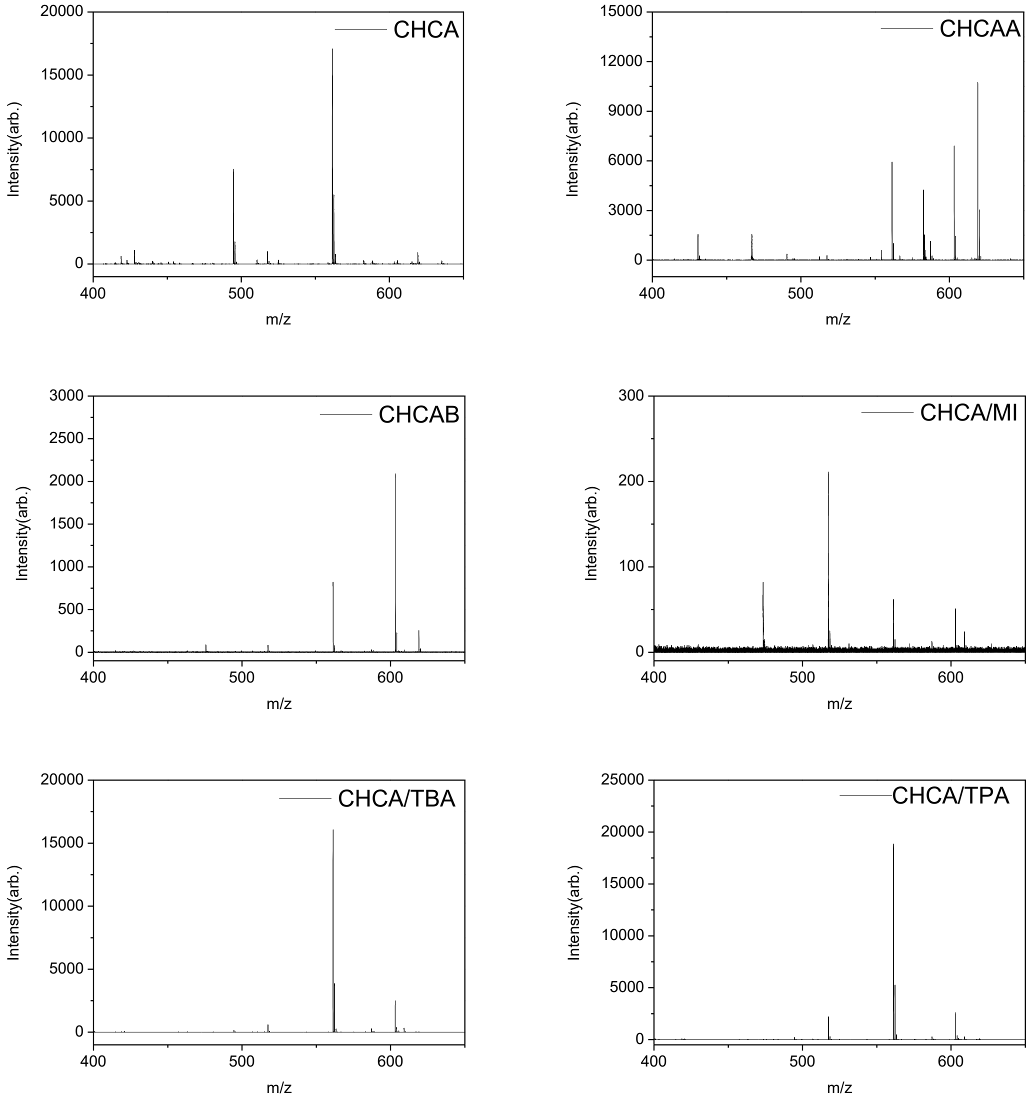

Fig S3. MALDI mass spectra of matrix background for (**a**) DHB ILs and (**b**) CHCA ILs in negative reflectron mode. Laser energy was 95% and 75% for each group. 1000 laser shots randomly distributed in 20 different positions.

(a) Negative DHB ILs

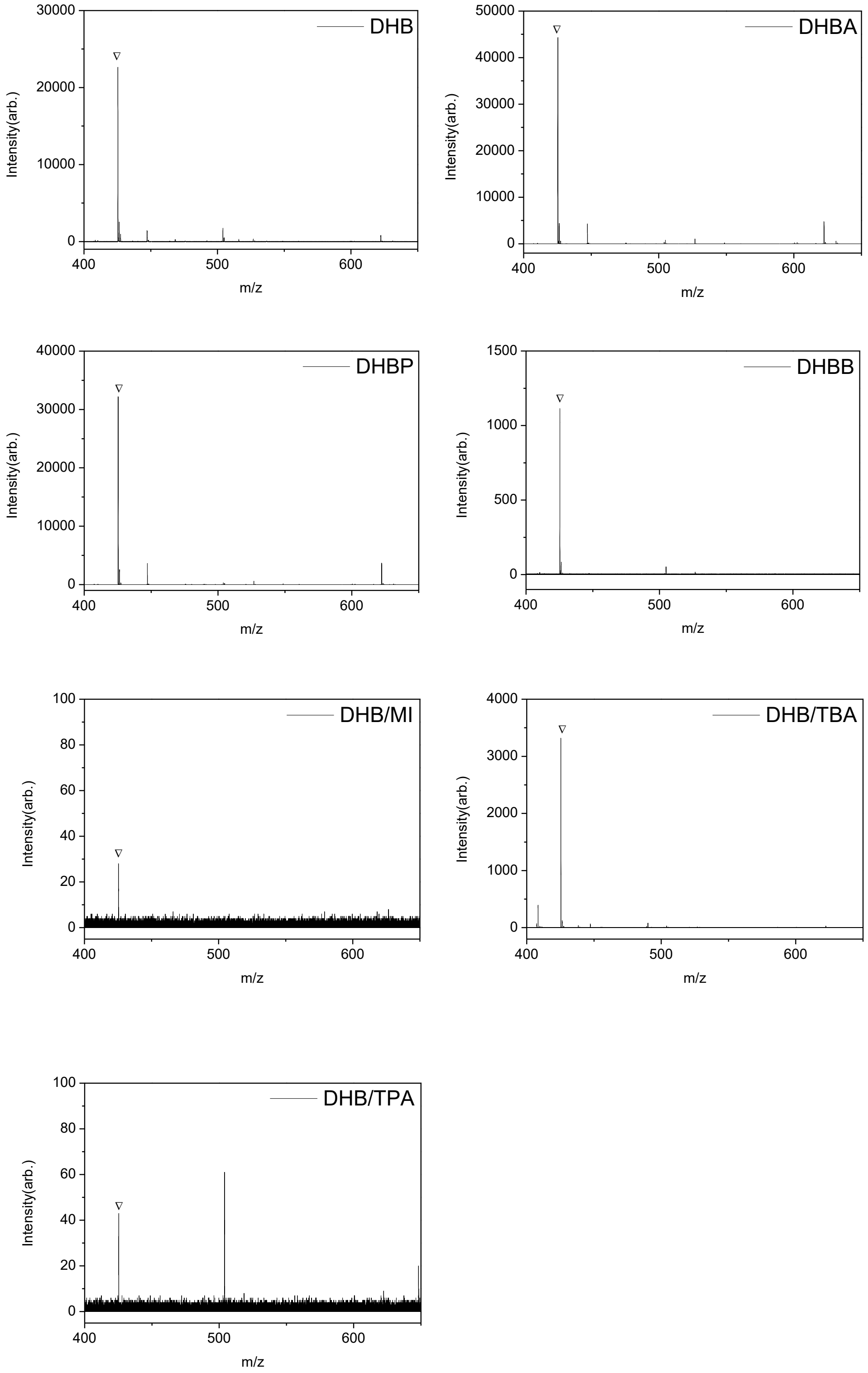

(b) Negative CHCA ILs

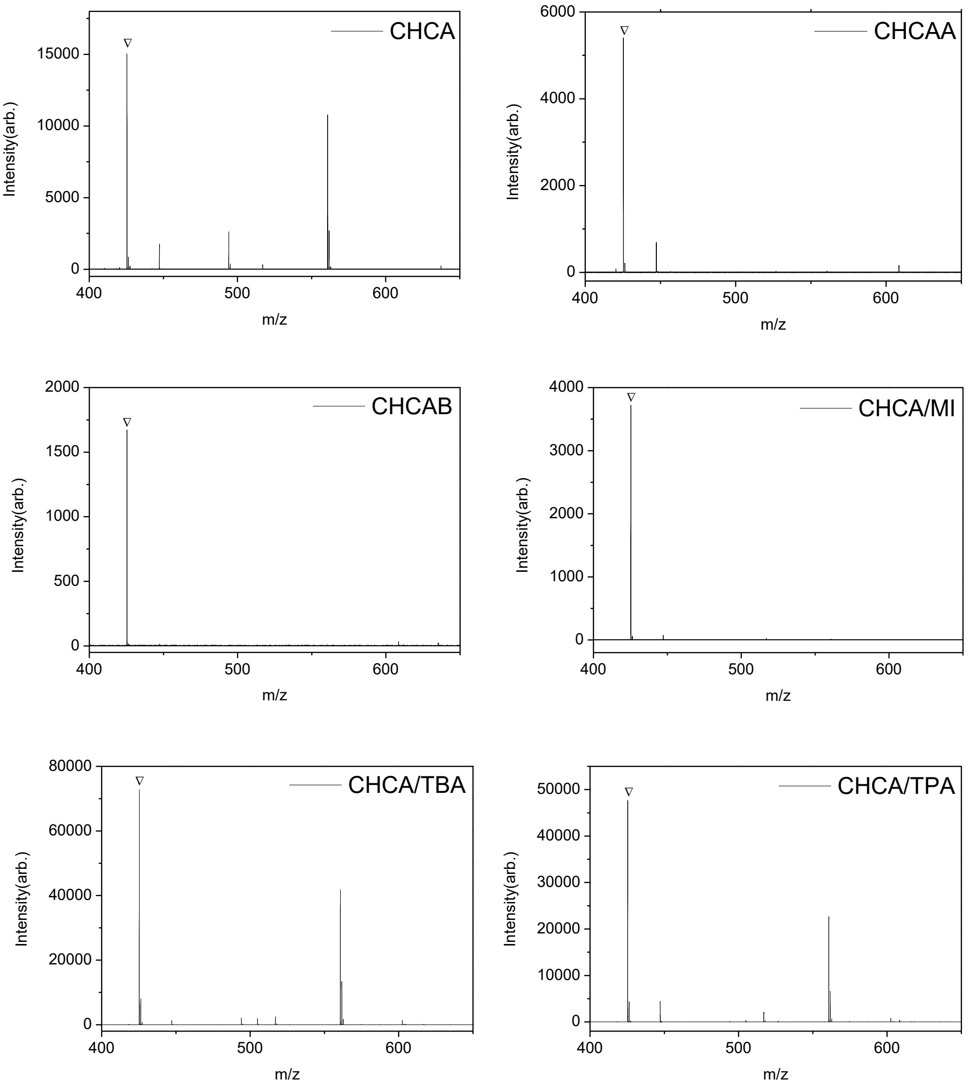

Fig S4. MALDI mass spectra of ADP (1 nmol) in one spot with the matrix of (**a**) DHB ILs and (**b**) CHCA ILs in negative reflectron mode. The molar matrix-to-analyte ratio was approximately 500:1. Laser energy was 95% and 75% for each group. 1000 laser shots randomly distributed in 20 different positions.

(a) Negative DHB ILs

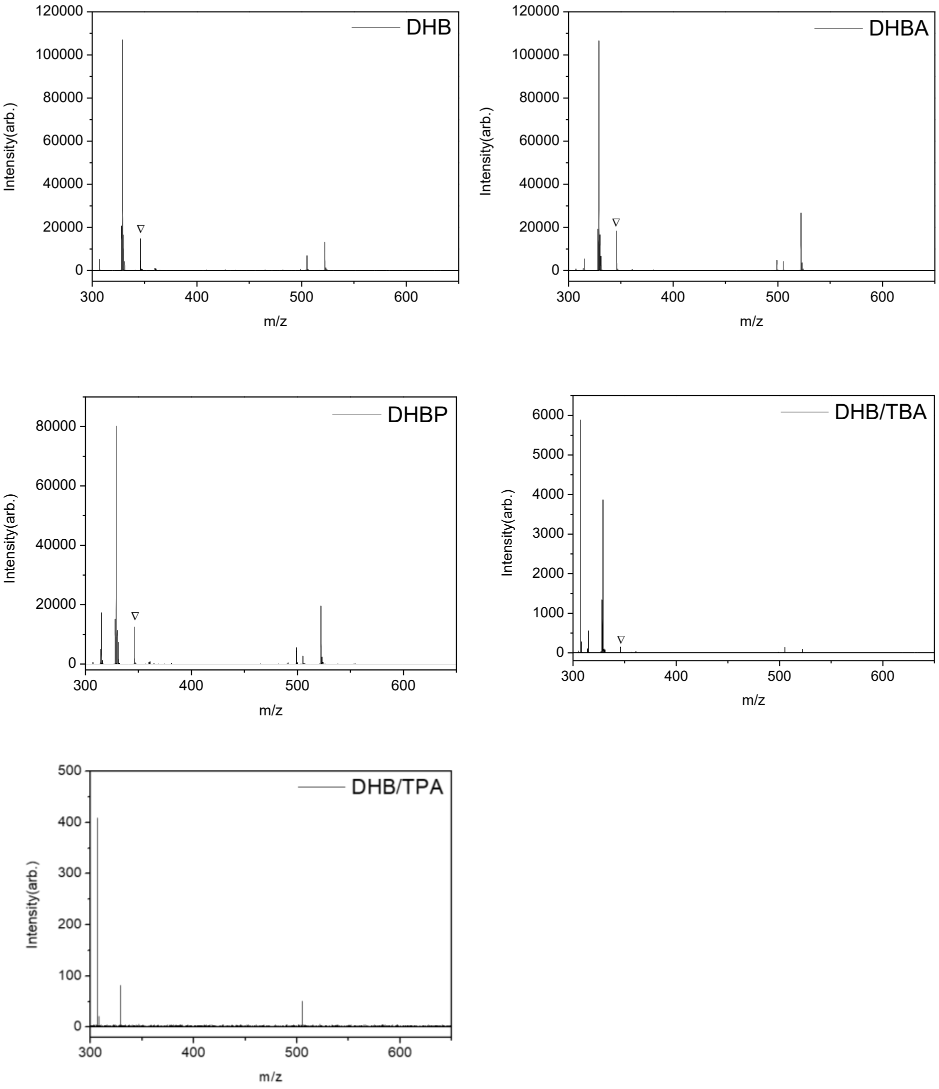

(b) Negative CHCA ILs

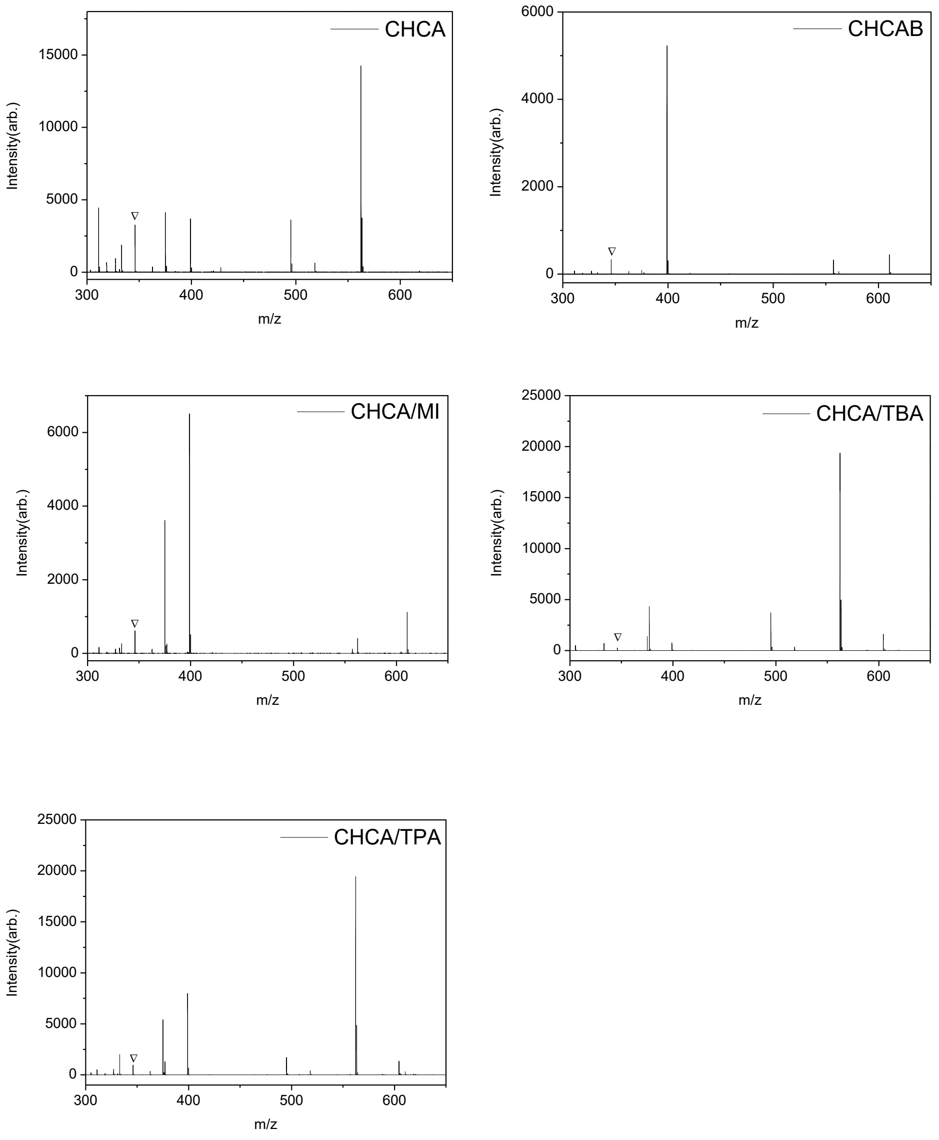

Fig S5. MALDI mass spectra of AMP (1 nmol) in one spot with the matrix of (**a**) DHB ILs and (**b**) CHCA ILs in negative reflectron mode. The molar matrix-to-analyte ratio was approximately 500:1. Laser energy was 95% and 75% for each group. 1000 laser shots randomly distributed in 20 different positions.

(a) DHB ILs

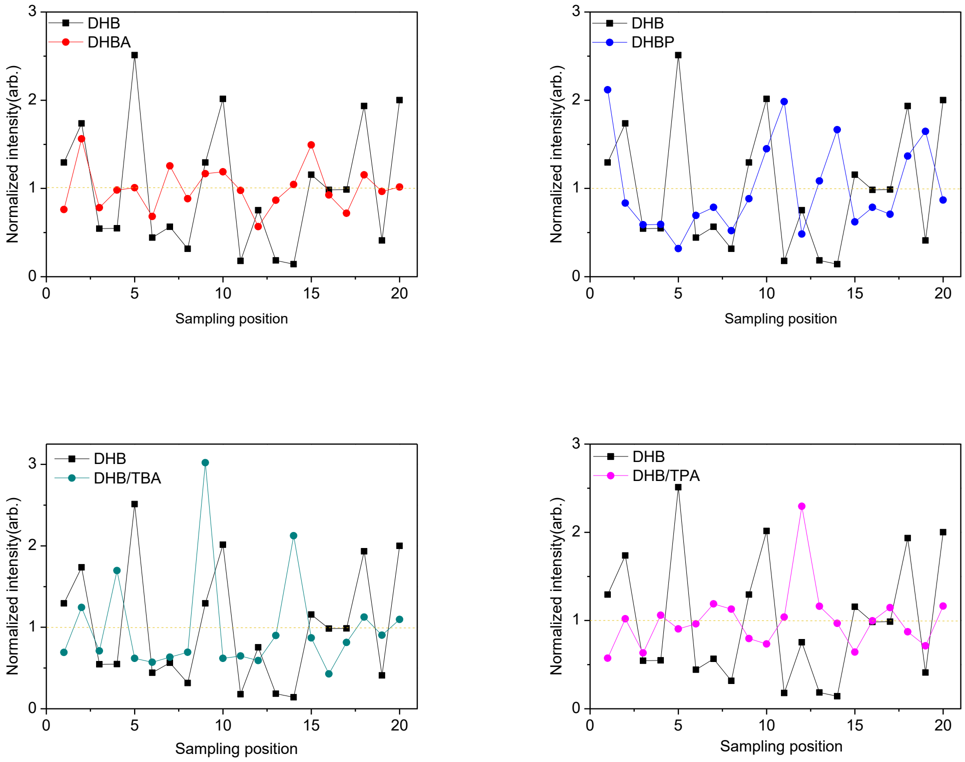
DHB
DHBA
DHBP
DHB/TBA
DHB/TPA
Normalized intensity(arb.)
Sampling position

(b) CHCA ILs

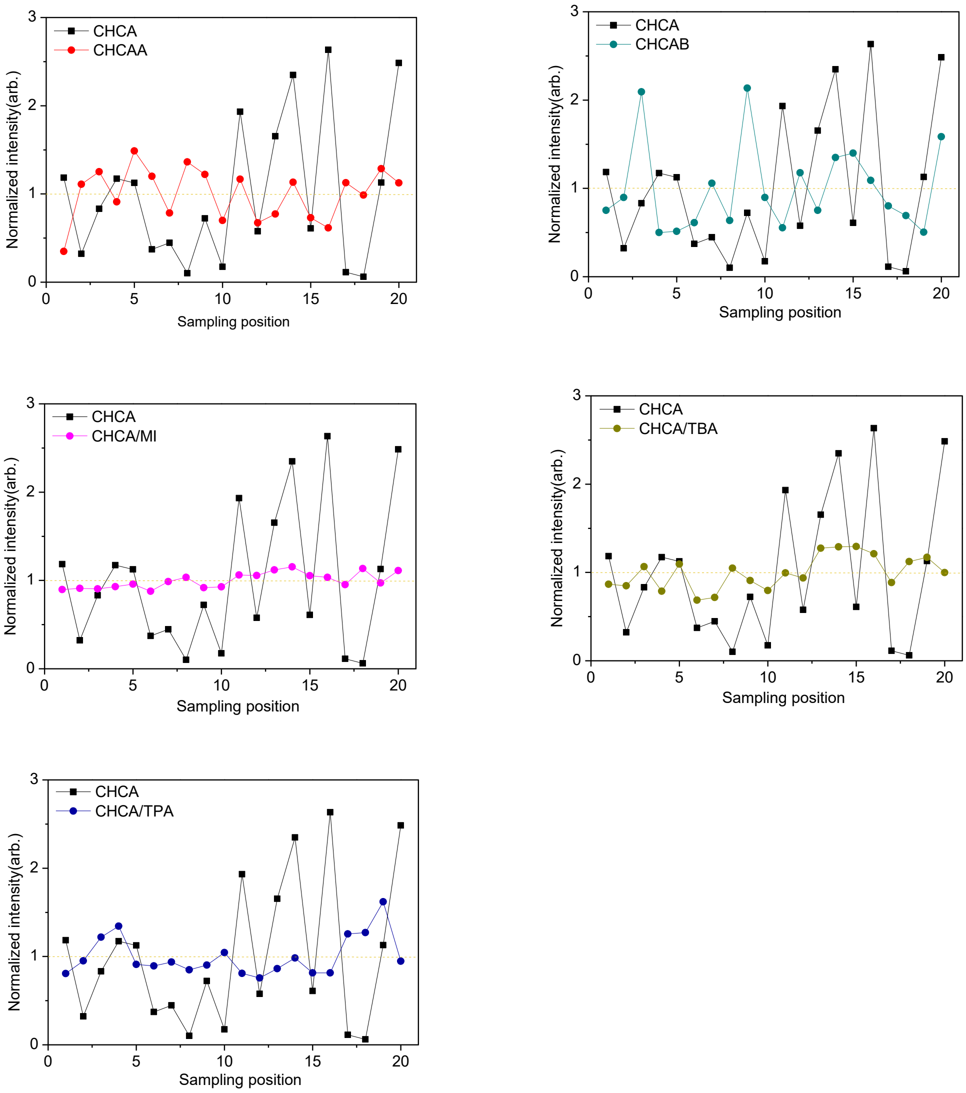

Fig S6. Plot of normalized [ATP-H]$^{-}$ signal distribution of (**a**) DHB ILs and (**b**) CHCA ILs in 20 different positions of one sample spot on the MALDI metal plate. Laser condition was 1000 shots in one point.

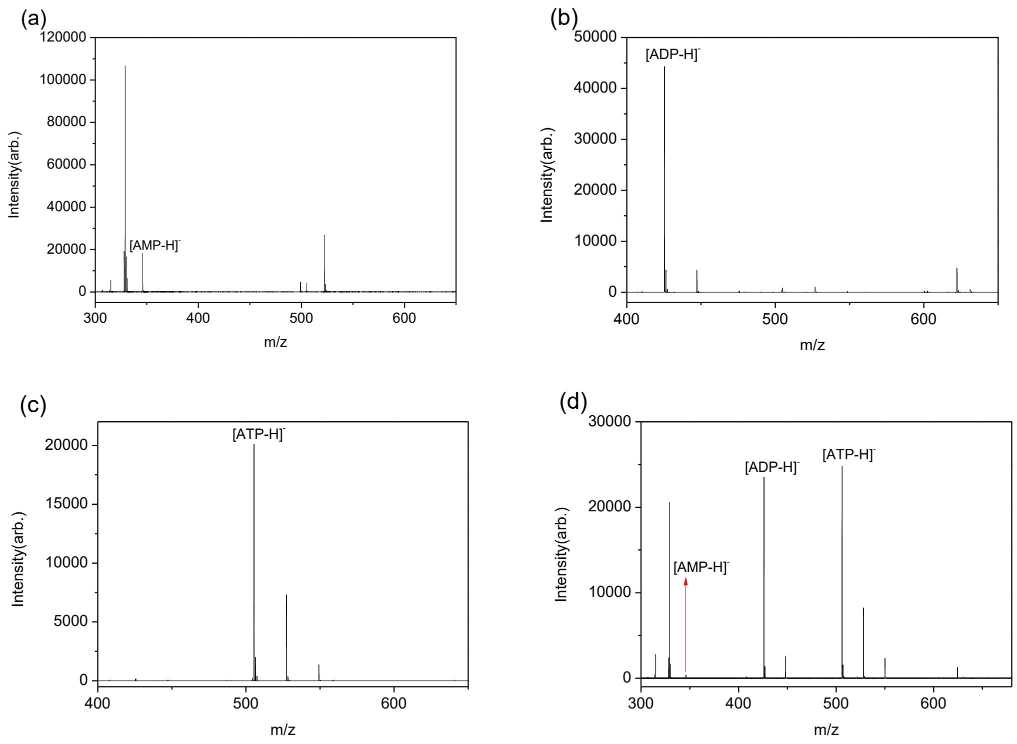

Fig S7. MALDI mass spectra of (a) AMP (1nmol) (b) ADP (1nmol) (c) ATP (1nmol) (d) AMP:ADP:ATP = 1:1:1 (0.33nmol for each one) with DHBA IL in negative reflectron mode. The molar matrix-to-analyte ratio was approximately 500:1. Laser intensity was 95%. 1000 laser shots randomly distributed in 20 different positions.

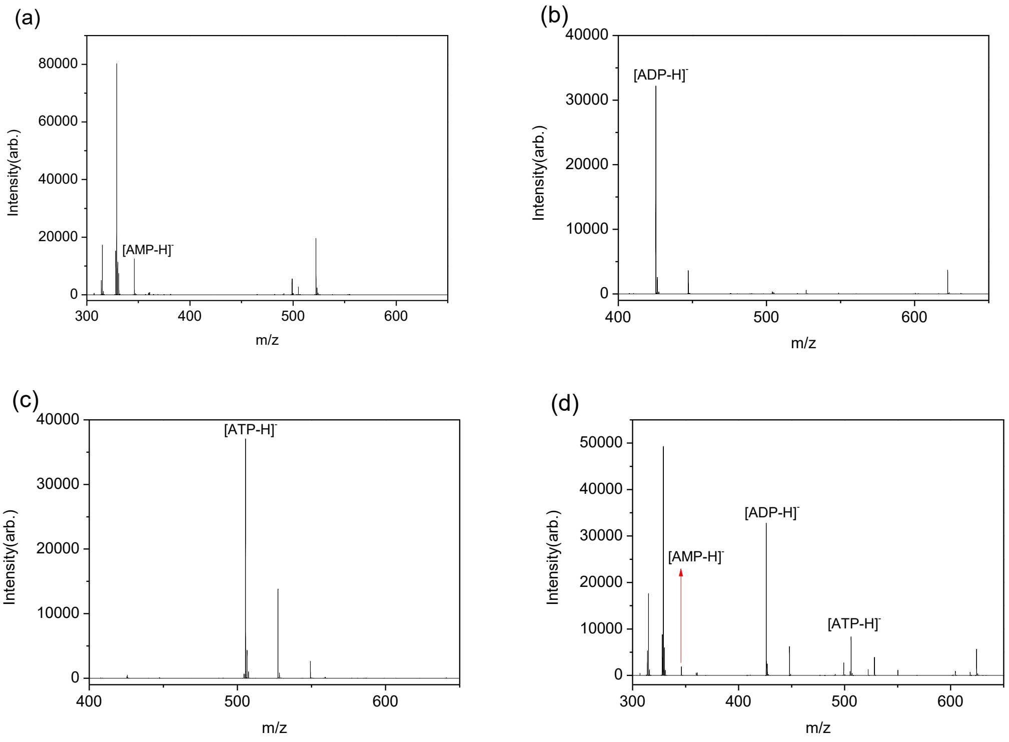

Fig S8. MALDI mass spectra of (a) AMP (1nmol) (b) ADP (1nmol) (c) ATP (1nmol) (d) AMP:ADP:ATP = 1:1:1 (0.33 nmol for each one) with DHBP IL in negative reflectron mode. The molar matrix-to-analyte ratio was approximately 500:1. Laser intensity was 95%. 1000 laser shots randomly distributed in 20 different positions.

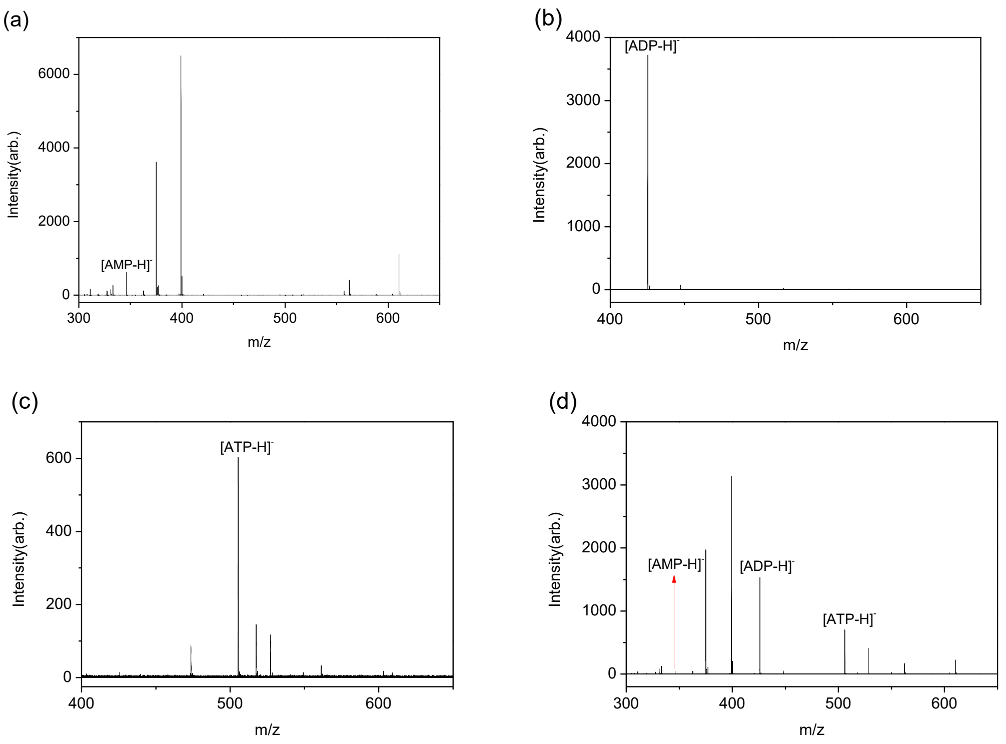

Fig S9. MALDI mass spectra of (a) AMP (1nmol) (b) ADP (1nmol) (c) ATP (1nmol) (d) AMP:ADP:ATP = 1:1:1 (0.33nmol for each one) with CHCA/MI IL in negative reflectron mode. The molar matrix-to-analyte ratio was approximately 500:1. Laser intensity was 75%. 1000 laser shots randomly distributed in 20 different positions.

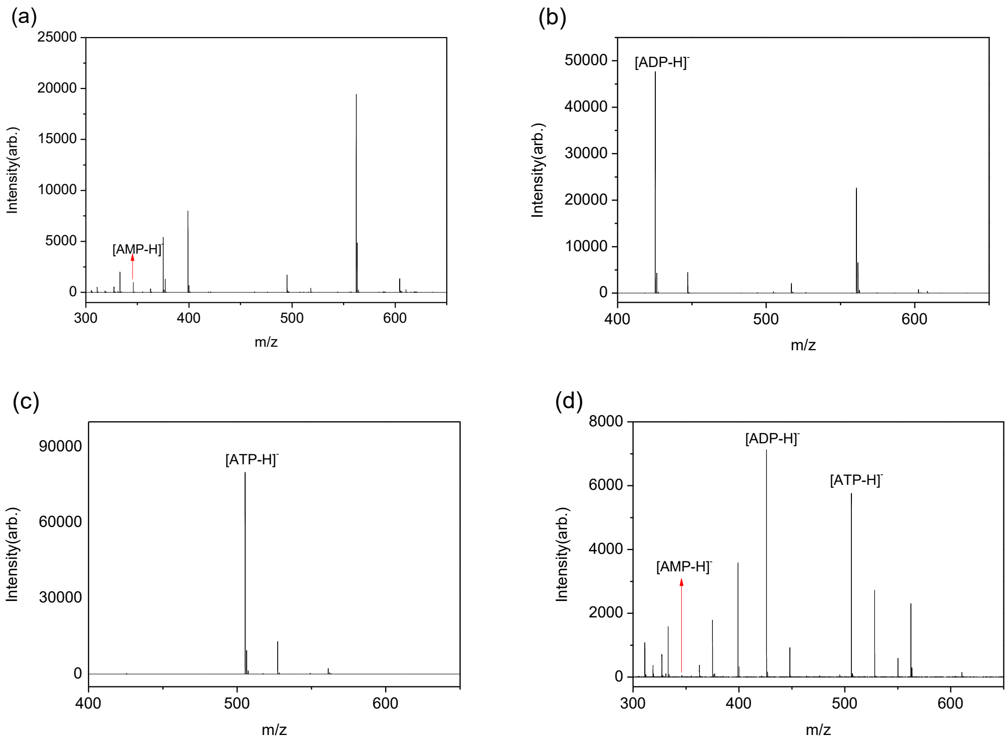

Fig S10. MALDI mass spectra of (a) AMP (1nmol) (b) ADP (1nmol) (c) ATP (1nmol) (d) AMP:ADP:ATP = 1:1:1 (0.33nmol for each one) with CHCA/TPA IL in negative reflectron mode. The molar matrix-to-analyte ratio was approximately 500:1. Laser intensity was 75%. 1000 laser shots randomly distributed in 20 different positions.

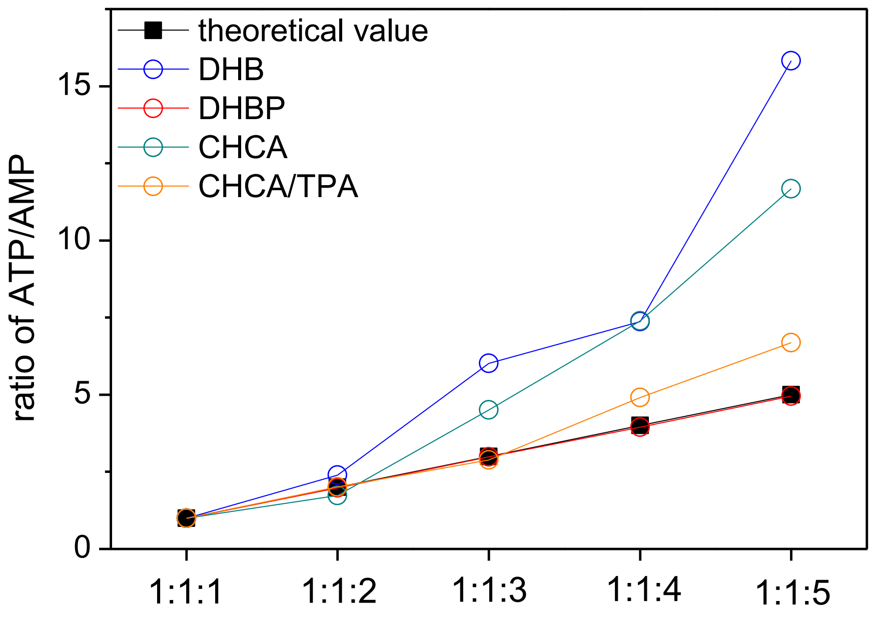

Fig S11. Quantitative comparison between theoretical and experimental ratios of AMP, ADP, and ATP. The molar ratio was adjusted from 1:1:1 until ATP had a concentration five times higher than that of AMP and ADP. The concentration of ATP was gradually increased, whereas concentrations of AMP and ADP were fixed. Normalizing signal intensities were calculated by 1:1:1 point: ratio of ATP/AMP from 1:1:1 to 1:1:5 (n = 8). 1000 laser shots randomly distributed in 20 different positions.

Table S1. Melting points of conventional matrices (DHB and CHCA) and IL matrices measured by the Mel-Temp II Capillary Melting Point Apparatus (Laboratory Devices Inc., USA).

	DHBA	DHBP	DHB/TBA	**DHB**
**mp (℃)**	74-76	82-84	94-96	204-206
	CHCAA	CHCAB	**CHCA**	
**mp (℃)**	85-87	98-100	245-247	
